# Alternative classification and screening protocol for transitional lumbosacral vertebra in German shepherd dogs

**DOI:** 10.1186/1751-0147-54-27

**Published:** 2012-05-01

**Authors:** Anu K Lappalainen, Reea Salomaa, Jouni Junnila, Marjatta Snellman, Outi Laitinen-Vapaavuori

**Affiliations:** 1 Section of Diagnostic Imaging, Department of Equine and Small Animal Medicine, Faculty of Veterinary Medicine, University of Helsinki, Helsinki, Finland; 2 Section of Small Animal Surgery, Department of Equine and Small Animal Medicine, Faculty of Veterinary Medicine, University of Helsinki, Helsinki, Finland; 3 4Pharma LTD, Turku, Finland

**Keywords:** Lumbosacral transitional vertebra, Radiographic screening, Dog, German shepherd dog

## Abstract

**Background:**

Lumbosacral transitional vertebra (LTV) is a common congenital and hereditary anomaly in many dog breeds. It predisposes to premature degeneration of the lumbosacral junction, and is a frequent cause of cauda equina syndrome, especially in German shepherd dogs. Ventrodorsal hip radiographs are most often used in diagnosis of LTV in screening programs. In this study, value of laterolateral lumbar spine radiographs as additions to ventrodorsal radiographs in diagnosis of LTV, and characteristics of LTV and the eighth lumbar vertebra (L8) in laterolateral radiographs were studied. Additionally, computed tomography (CT) features of different types of LTV were elucidated.

**Methods:**

The ventrodorsal pelvic and laterolateral lumbar spine radiographs of 228 German shepherd dogs were evaluated for existence and type of LTV. Morphology of transverse processes was used in classification of LTV in ventrodorsal radiographs. The relative length of sixth (L6) and seventh (L7) vertebrae (L6/L7) was used in characterization of these vertebrae in laterolateral radiographs. CT studies were available for 16 dogs, and they were used for more detailed characterization of different types of LTV. Non-parametric *χ*^2^ statistics, generalized logit model for multinomial data, and one-way analysis of variance was used for statistical analyses.

**Results:**

In all, 92 (40%) dogs had a LTV, the most common type being separation of first spinous process from the median crest of the sacrum in 62 dogs (67% of LTV). Eight dogs had eight lumbar vertebrae. Those dogs with LTV had longer L7 in relation to L6 than dogs with normal lumbosacral junctions. When L6/L7 decreased by 0.1 units, the proportion of dogs belonging to the group with L8 was 14-fold higher than in the group with normal lumbosacral junctions. L8 resembled first sacral vertebra (S1) in length and position and was therefore classified as one type of LTV. With CT it was shown that categorizing LTV, based on shape and visibility of transverse processes seen in ventrodorsal radiographs, could be misleading.

**Conclusions:**

We suggest that L8 be included as a part of the LTV complex, and the laterolateral radiographs of the lumbar spine be considered as an addition to ventrodorsal projections in the screening protocols for LTV.

## Background

The sacrum consists of three fused vertebrae and develops, as do the other vertebrae, from three primary ossification centers. The first and second segments have additional ossification centers laterally. They represent ancestral forms of ribs and develop into the ventral parts of the sacral wings [1]. Lumbosacral transitional vertebra (LTV) is a common congenital anomaly seen in several dog breeds [2-4]. LTV predisposes to premature degeneration of the lumbosacral junction, is a frequent cause of cauda equina syndrome, especially in German shepherd dogs [4,5], and is thought to be hereditary [6].

The definition of LTV varies in the veterinary literature. It has been described as a vertebra having features of both lumbar and sacral vertebrae, in which a disc space exists between the first (S1) and second (S2) sacral segments [3]. Several classification systems of LTV, based on the morphology of the sacral wings and their attachment to the ilium, have been used. It can be classified, based on the morphological characteristics of the transverse processes and their relationship to the ilium seen in ventrodorsal radiographs [6], or based on alterations of the costal processes (this is synonymous with the transverse process used commonly in veterinary literature) and ventral alar elements [7]. In a recent study, LTV was divided into five categories, based on ventrodorsal radiographs, in which type 1 was a normal sacrum, type 2 was otherwise normal, but with separation of the S1 spinous process from the median crest, and types 3–5 were more explicit forms of symmetrical and asymmetrical alterations in the transverse processes [8]. A small number of German shepherd dogs have shown an eighth lumbar vertebra (L8), but this has not been taken into account in the classification of LTV [6,8]. However, it seems possible that the presence of L8 is part of the LTV complex [9].

LTV is usually diagnosed radiographically, using a ventrodorsal projection [6-8,10], but the laterolateral projection has been used in combination with ventrodorsal to obtain a diagnosis [3]. Computed tomography (CT) is widely used in diagnostics of lumbosacral disease [11] and with this modality the sacrum can be seen without superimposition of other pelvic structures. The appearance of the different types of LTV has not been described in detail using CT.

The prevalence of LTV in German shepherd dogs is reported to be from 4.3% to 29.0% [6,8,10] and is thought to be hereditary [6,8]. Due to the hereditary base as well as orthopedic and neurological consequences caused by LTV, use of affected dogs for breeding is discouraged [2]. Screening for LTV in addition to hip dysplasia is possible, since the lumbosacral junction is visible in hip-screening radiographs [12]. However, an LTV is not always visible in ventrodorsal radiographs [4].

The aims of this study were to determine the diagnostic value of the laterolateral radiographic projection as an addition to ventrodorsal projection in diagnosing LTV in screening programs for German shepherd dogs, and to describe the features of LTV and L8 in laterolateral radiographs. An additional aim was to compare radiographic and CT features of different types of LTV. Our hypotheses were that L8 is part of the LTV complex, and that a laterolateral radiographic projection increases significantly the accuracy of the diagnosis of LTV in screening programs.

## Methods

The ventrodorsal pelvic and laterolateral lumbar spine radiographs of German shepherd dogs were collected from three sources: 1) Finnish Kennel Club hip dysplasia screening radiographs in 2007, when an additional laterolateral projection of the lumbar spine, as requested by the national breed club, was included in the screening radiographs, 2) radiographs from the Finnish Border Guard of dogs (born 1995–2006) withdrawn from active service, and 3) radiographs and CT studies collected from the database of the Veterinary Teaching Hospital of the University of Helsinki between May 2005 and March 2011 of dogs radiographed for various reasons. A CT study of the lumbosacral area was included in the study when available.

**Figure 1:**
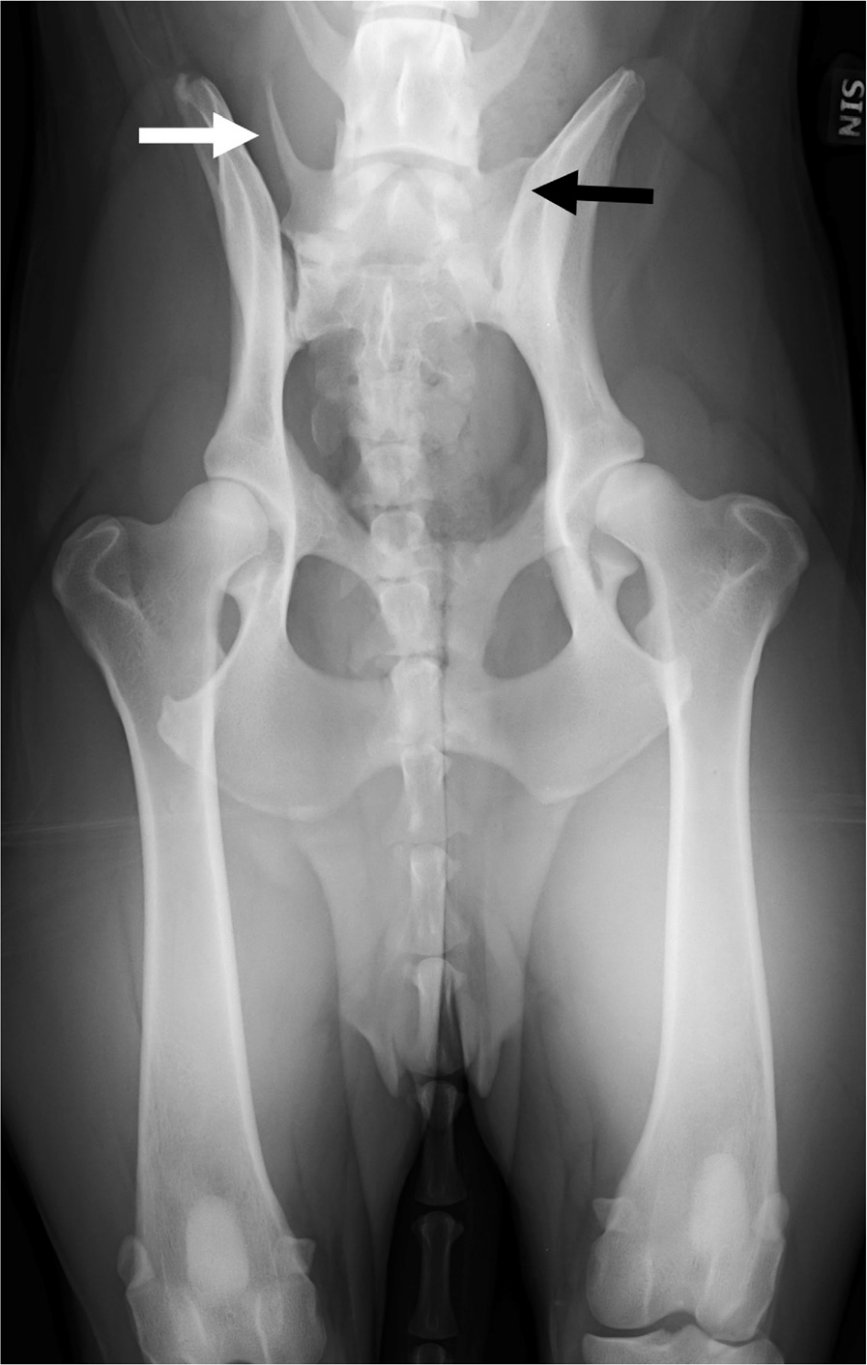
A ventrodorsal radiograph of a German shepherd dog with lumbosacral transitional vertebra of type 1 on the right side (white arrow) and type 3 on the left side (black arrow).

**Figure 2:**
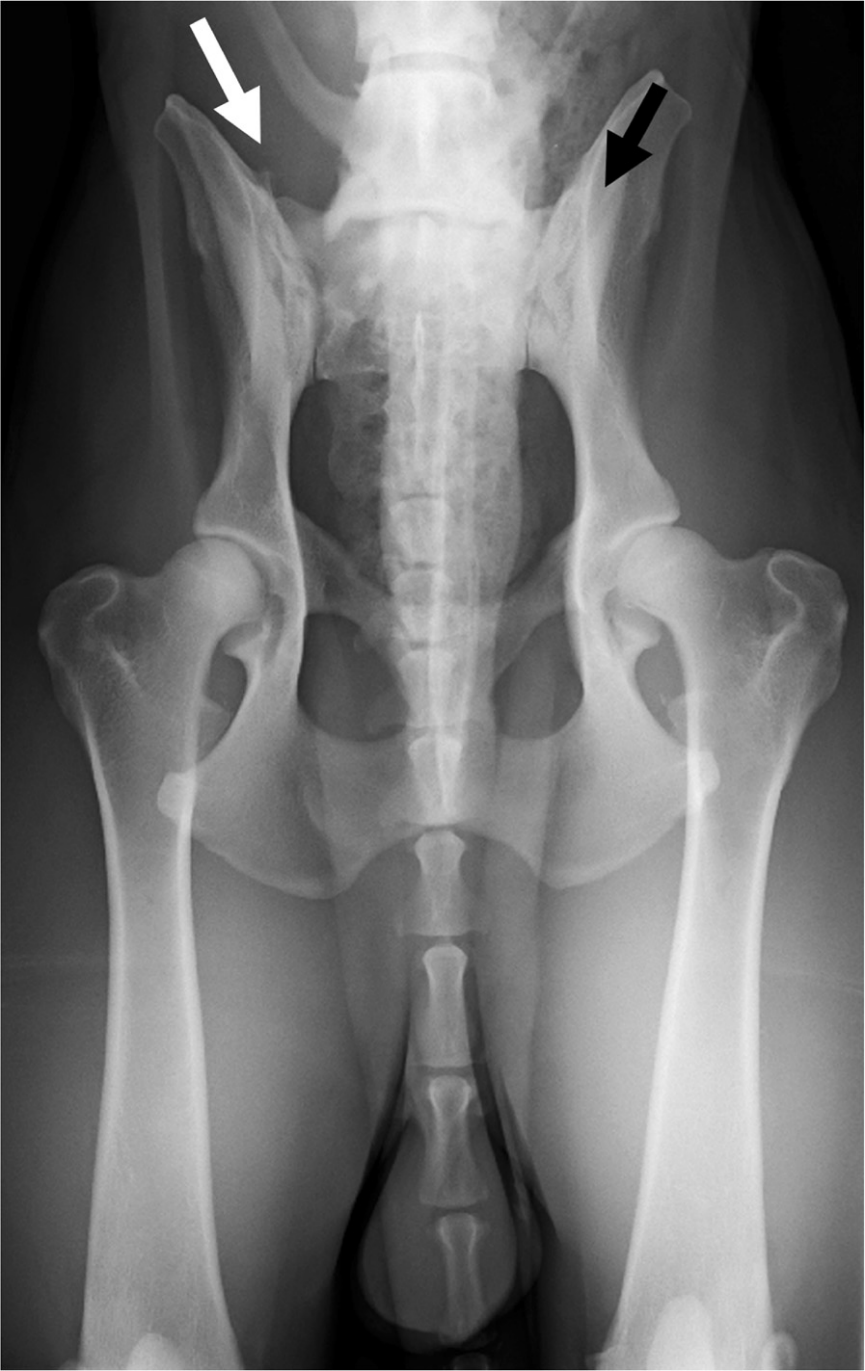
**A ventrodorsal radiograph of a German shepherd dog with lumbosacral transitional vertebra of type 2 on the right side (white arrow) and type 3 on the left side (black arrow).** On the right side the tip of the transverse process is visible, on the left side it is not.

**Figure 3:**
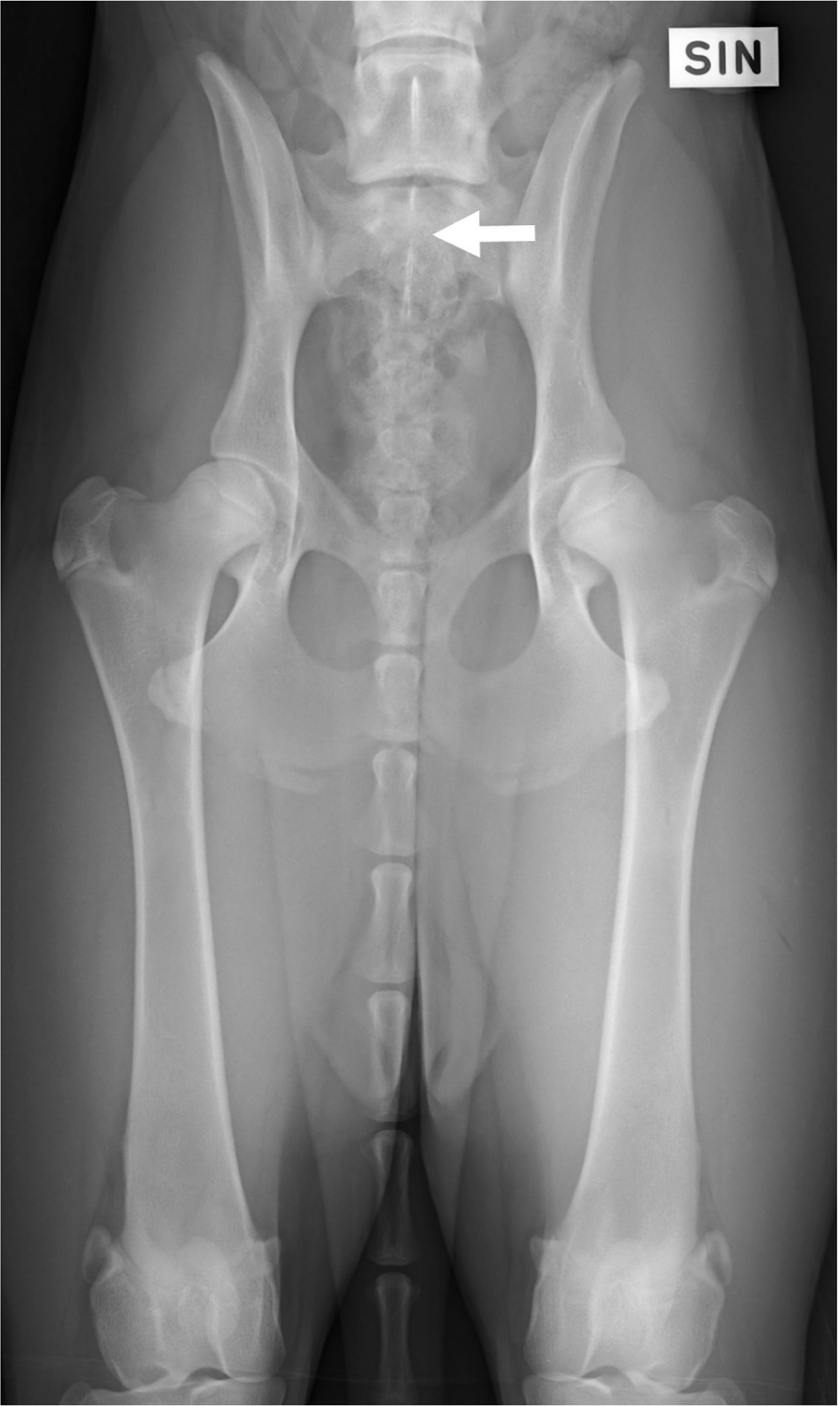
A ventrodorsal radiograph of a German shepherd dog with separation of S1 from the median crest of the sacrum (arrow).

**Figure 4:**
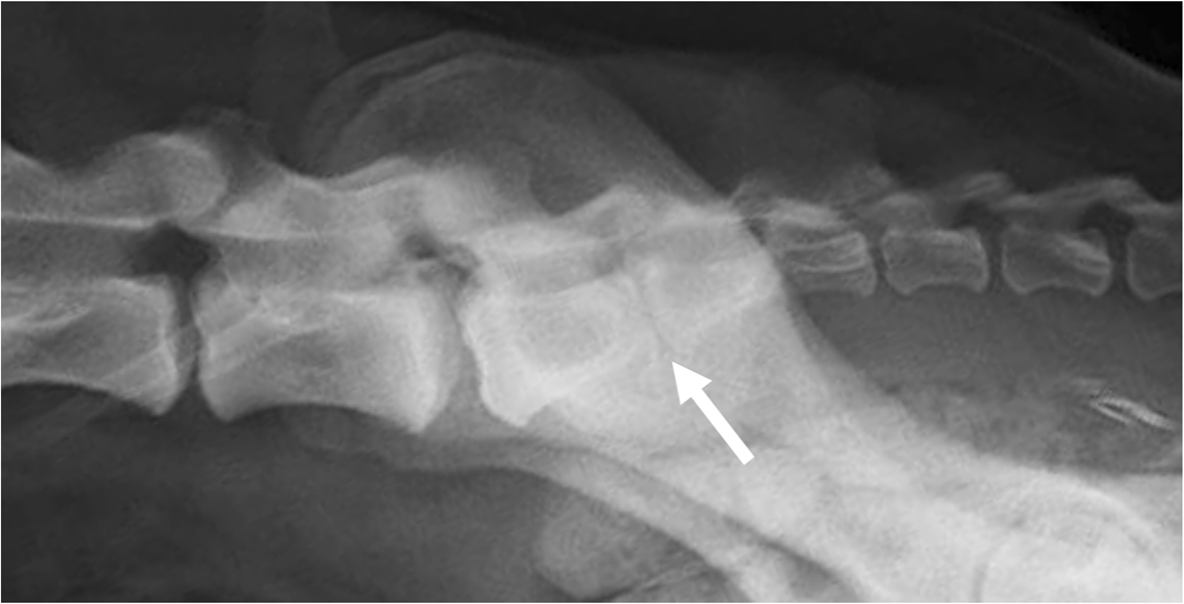
**A laterolateral radiograph of a German shepherd dog with a lumbosacral transitional vertebra of type 3 on the left and type 2 on the right side.** A radiolucent line is visible between S1 and S2.

Two of the authors evaluated the ventrodorsal and laterolateral radiographs for the existence and type of LTV independently, blinded to the findings of the other projection. Thereafter, the two authors evaluated both projections together, and a diagnosis of LTV was based on consensus. An LTV seen in the ventrodorsal projection was diagnosed and classified into three types based on the morphology of the transverse processes, according to the previously published scheme [6]. In type 1 (lumbar type), the transverse processes resembled the lumbar processes (Figure 1), in type 2 (intermediate type) they were partly superimposed on the ilium, but the tip of the process appeared free (Figure 2), and in type 3 (sacral type) the processes were totally superimposed on the ilium or resembled the sacral vertebra (Figures 1 and 2). Each type was further divided into symmetrical and asymmetrical subcategories. The radiolucent space between the S1 and S2 spinous processes (separation of S1 spinous process from the median crest of the sacrum) was recorded in the ventrodorsal projection (Figure 3) and this was named as type 4. In the laterolateral radiographs, the diagnosis of LTV was based on the visibility of a radiolucent line between S1 and S2 (Figure 4). Dogs with L8 were included in the LTV group.

**Figure 5:**
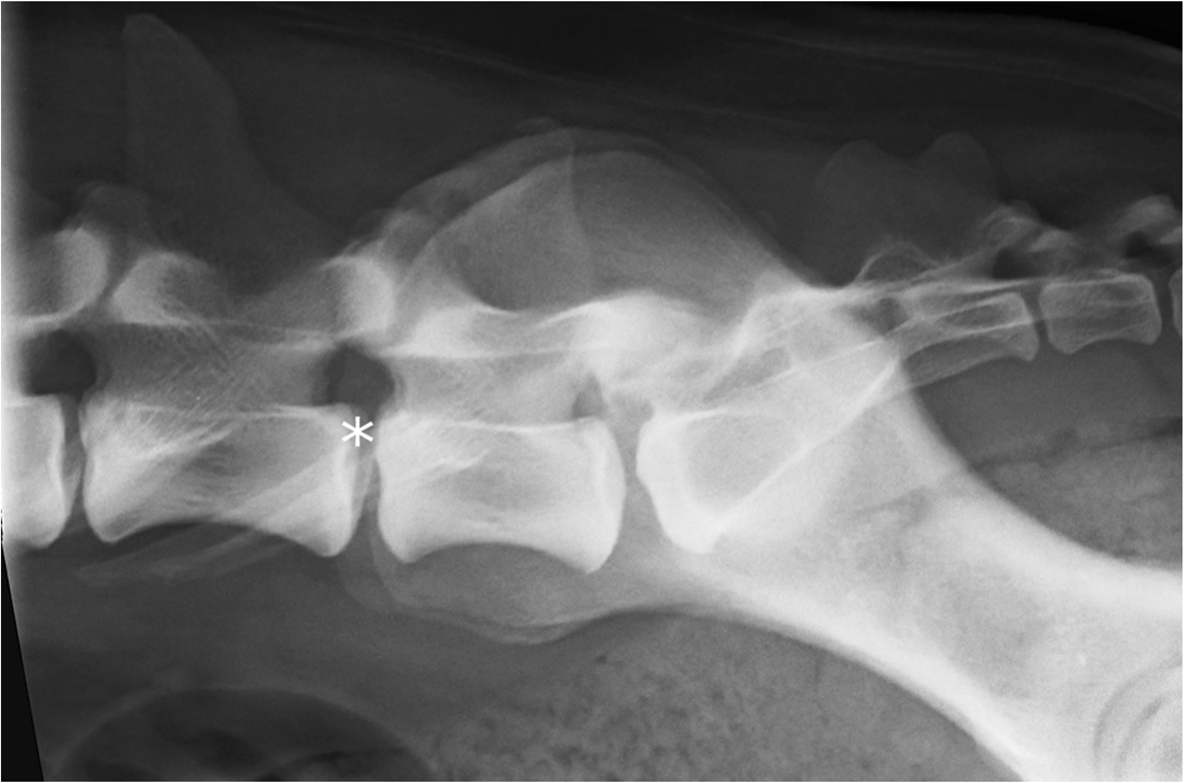
**A laterolateral radiograph of lumbosacral area of a German shepherd dog with radiographically normal lumbosacral junction.** The cranial borders of the iliac bones are superimposed on the L6/L7 disc space (asterisk).

**Figure 6:**
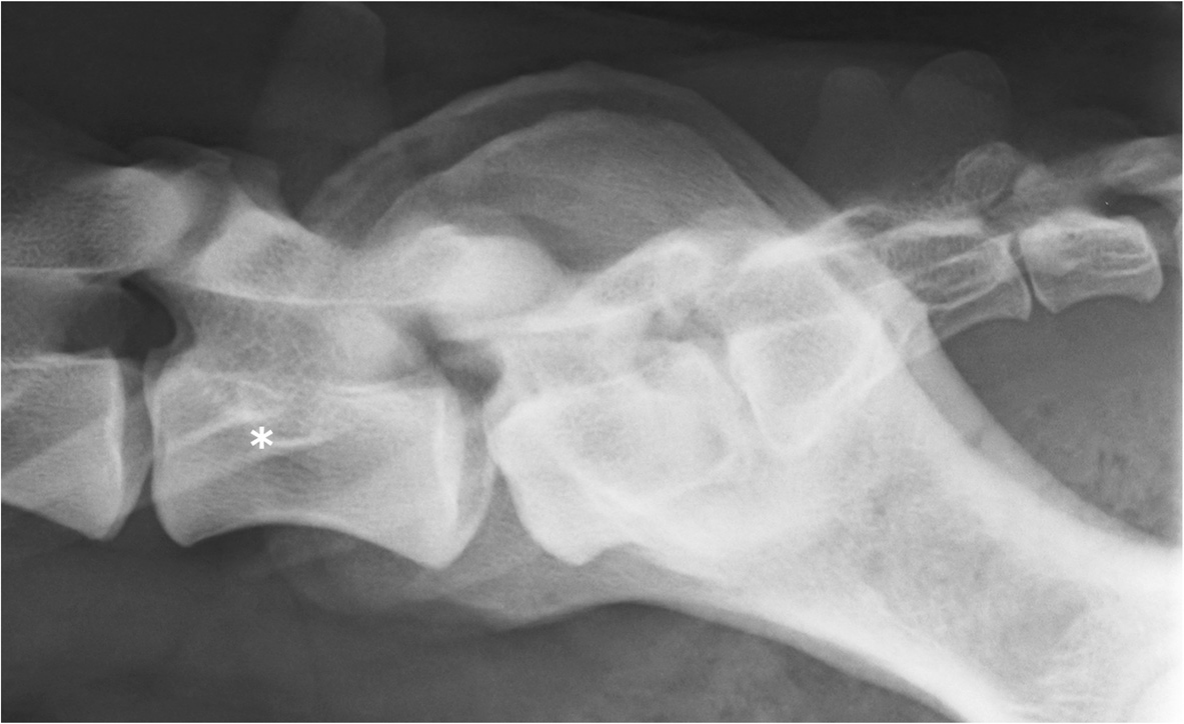
**A laterolateral radiograph of lumbosacral area of a German shepherd dog with a lumbosacral transitional vertebra.** The cranial borders of the iliac bones are superimposed on the cranial half of L7 (asterisk).

**Figure 7:**
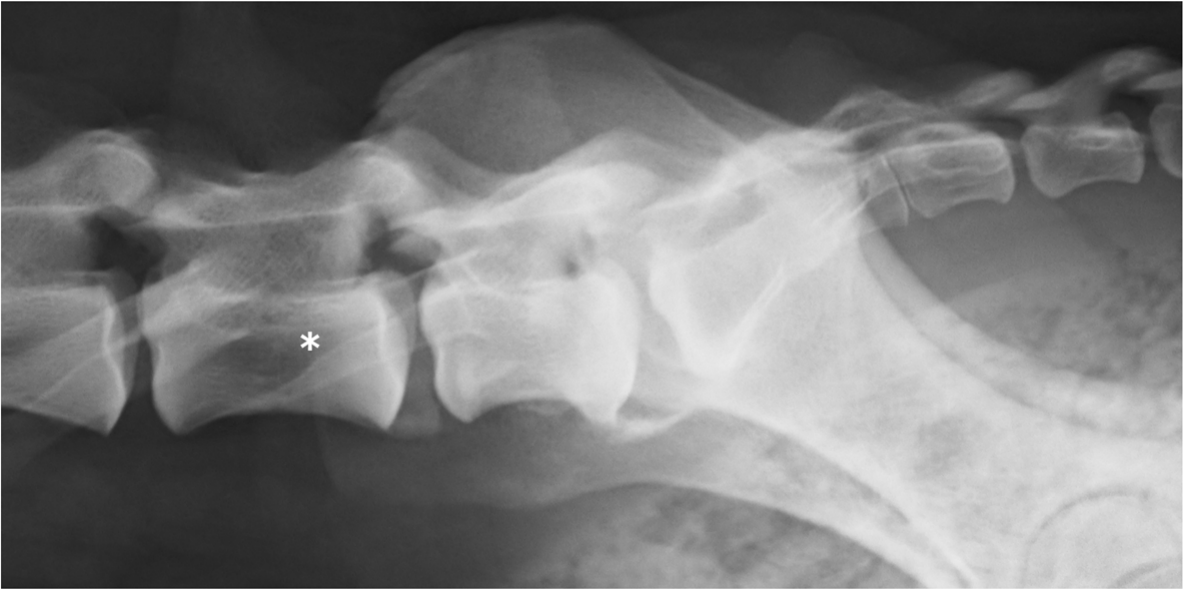
**A laterolateral radiograph of lumbosacral area of a German shepherd dog with a lumbosacral transitional vertebra with eight lumbar vertebrae.** The cranial borders of the iliac bones are superimposed on the caudal half of L7 (asterisk).

**Figure 8:**
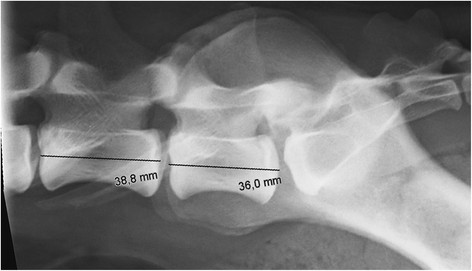
A laterolateral radiograph of the caudal lumbar spine of a normal German shepherd illustrating measurement of midbody length of L6 and L7.

The number of lumbar vertebrae was counted using the last thoracic vertebra as a reference point. The first lumbar vertebra (L1) was the vertebra caudal to this vertebra. To evaluate the appearance of the last presacral vertebra, the laterolateral radiographs were evaluated for position of seventh and length of sixth and seventh (L6 - L7) vertebrae (and L8 when present). The position of L7 relative to the ilium was recorded. It was graded as caudal if the cranial border of the ilium was cranial to the cranial endplate of L7 (Figure 5), intermediate if the cranial border of the ilium was superimposed on the cranial half of L7 (Figure 6), and cranial if the cranial border of the ilium was superimposed on the caudal half of L7 (Figure 7). Additionally, the midcorpus length (mm) of L6 and L7 (Figure 8) was measured with a ruler and the relative length was calculated from a formula (length of L6/length of L7) to remove the effect of size of the dog. In dogs with eight lumbar vertebrae, the length of L8 was measured with a ruler, relative length was calculated from a formula (length of L7/length of L8) and its position relative to the ilium was recorded.

**Figure 9:**
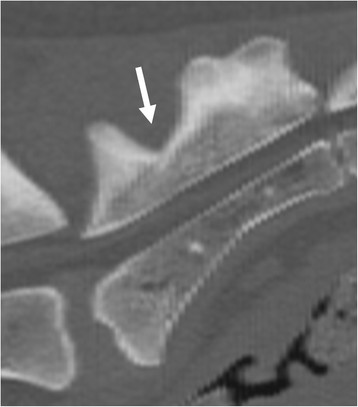
A CT midplane sagittal reconstruction image of the same dog as in Figure 3 with separation of the first sacral spinous process from the median crest of the sacrum (arrow).

**Figure 10:**
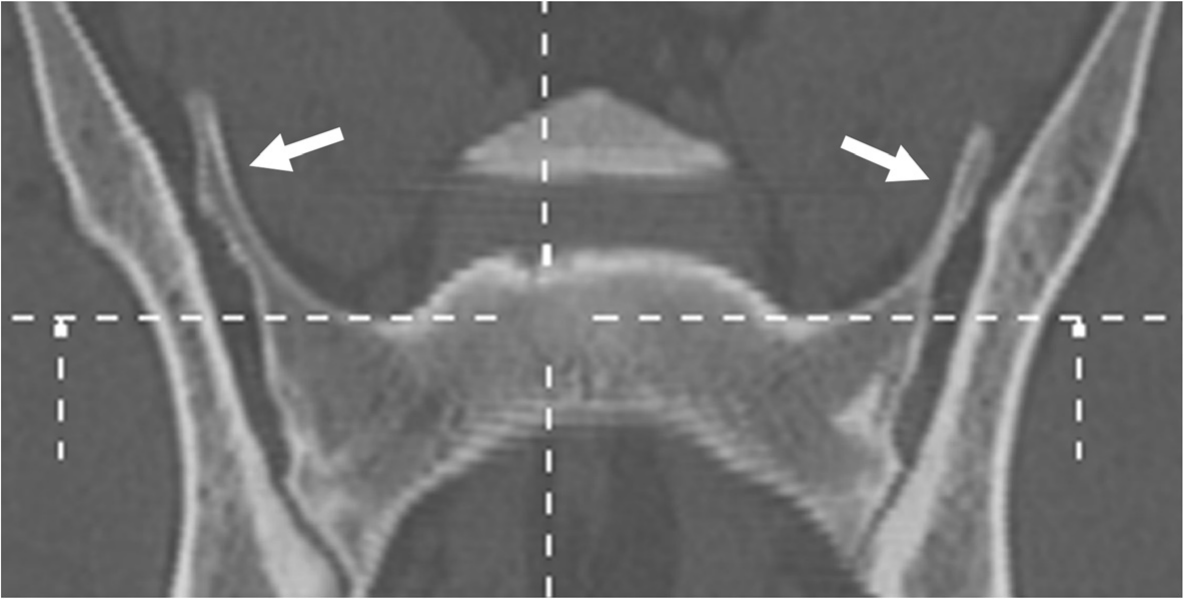
**A CT dorsal reconstruction image of a German shepherd dog radiographically diagnosed as symmetrical LTV of lumbar type.** Bilateral transverse processes are evident (arrows).

**Figure 11:**
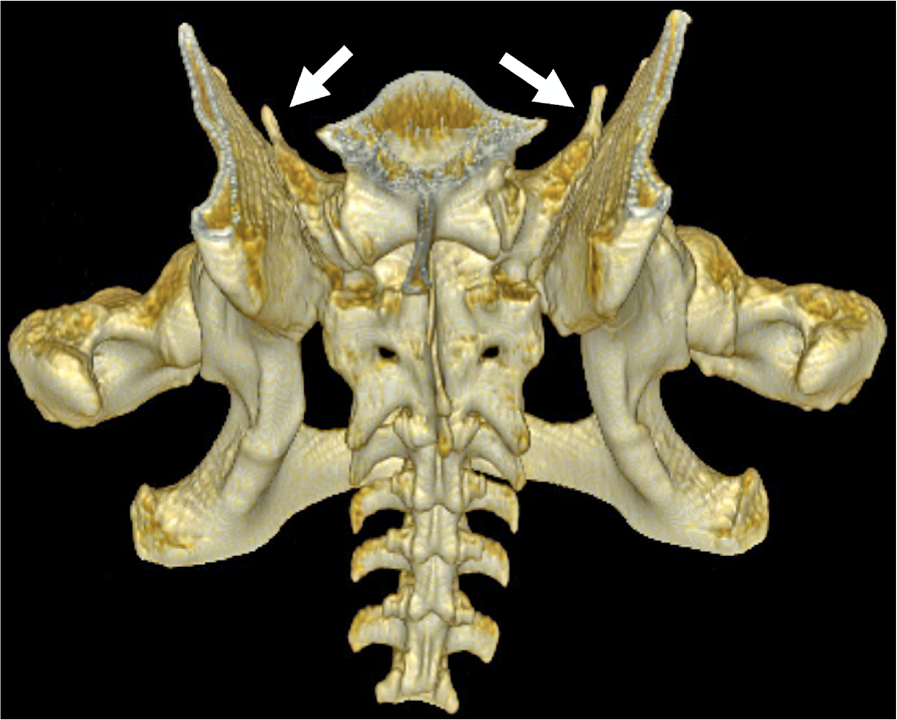
**A CT volume-rendering technique image of the same dog as in Figure 10.** This dog had a deep indentation between S1 and S2 but the vertebral bodies were fused. The tips of the transverse processes (arrows) were superimposed by the iliac wings in the ventrodorsal radiograph.

The primary author evaluated the CT images. Multiplanar reconstructions (MPR) (Figures 9 and 10) and volume-rendering technique (VRT) (Figure 11) were used in assessment. The shape of the sacrum, including the shape of the median crest, was compared with the appearance of the sacrum in ventrodorsal radiographs.

The sensitivity was calculated for the ventrodorsal radiographic projection in diagnosis of LTV with a non-parametric *χ*^2^ statistics. A one-sided p-value was used to determine the statistical significance of the difference.

The relationship between type of lumbosacral junction and relative length of L6/L7 was investigated with generalized logit model for multinomial data. The model included type of the lumbosacral junction as the response variable and the relative length of L6/L7 as a fixed effect. In the model, the differences between groups were quantified with odds ratios and 95% confidence intervals (CI). Dogs with normal lumbosacral junctions based on radiographs were used as the reference category. Since there were very few or no observations in many cells of the location of the L7 type, a generalized logit model could not be fitted to the data properly. Thus, only descriptive statistics of the relationship between the type of lumbosacral junction and location of L7 are provided. The difference between types of lumbosacral junction was analysed with one-way analysis of variance. Here, the response variable was the relative length of L6/L7 and the type of lumbosacral junction was used as a fixed factor. Statistical significance was set at the 5% level. SAS System for Windows version 9.2 (SAS Institute Inc. Cary NC, USA) was used for all statistical calculations.

## Results

In all, 228 German shepherd dogs were included in the study, of which 79 were males and 149 were females. The mean age of the dogs was 42 months (range 5 months - 134 months). LTV was diagnosed in 92 dogs (40.4%), based on ventrodorsal and/or laterolateral radiographs. Ten dogs with eight vertebrae were included in the LTV group. In Six of these dogs, the only abnormal radiographic finding was a short and caudally positioned L8. In addition four dogs had L8 and nonfused S2-S3.

Results regarding the type of LTV are presented in Table 1. LTV was diagnosed from both projections in 15 cases (16.3%) from only the ventrodorsal radiograph in 62 cases (67.4%) and from only the laterolateral radiograph in ten cases (8.7%). All cases diagnosed, based only on the ventrodorsal projection, had normally fused sacral bodies. All dogs with eight lumbar vertebrae were diagnosed by laterolateral projection only. There was a statistically significant improvement (p = 0.003) detected in the sensitivity of the diagnosis when the laterolateral projection was used together with the ventrodorsal projection compared with the ventrodorsal projection alone. The sensitivity rose from 90% to 100%.

**Table 1:** Types of lumbosacral transitional vertebrae seen in laterolateral and ventrodorsal radiographic projections in 92 German shepherd dogs

Type of LTV	VD	LAT	VD and LAT	Total
3/3 (left/right)	4		1	5
2/2 (left/right)	1			1
3/2, 3/1 or 2/1 (left/right)			14	14
4	62			62
L8		10	0	10
Total	67	10	15	92

LTV = lumbosacral transitional vertebra, VD = ventrodorsal projection,

LAT = laterolateral projection, 3 = sacral type, 2 = intermediate type,

1 = lumbar type, 4 = separation of S1 spinous process from the median crest,

L8 = eighth lumbar vertebra.

**Figure 12:**
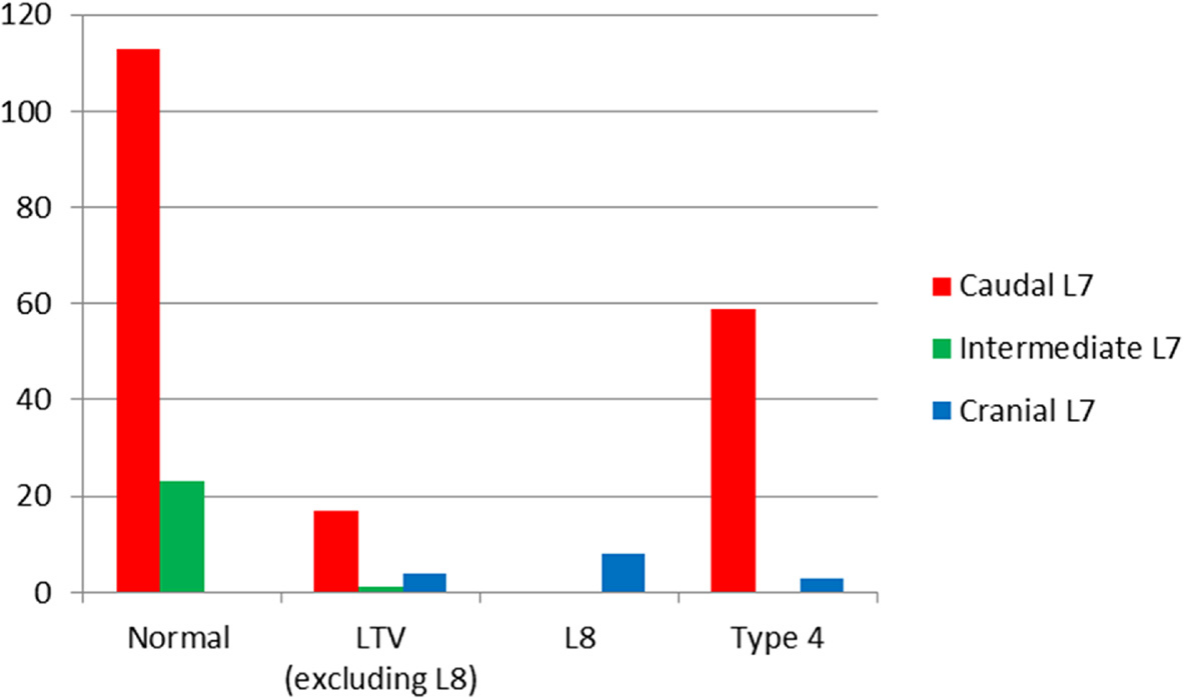
**The position (caudal, intermediate or cranial) of L7 in relation to the ilium in 228 German shepherd dogs with different types of lumbosacral junctions.** Normal = normal lumbosacral junction, Type 4 = separation of S1 spinous process from the median crest of the sacrum, LTV = lumbosacral transitional vertebra of types 1–3, L8 = eighth lumbar vertebra.

**Figure 13:**
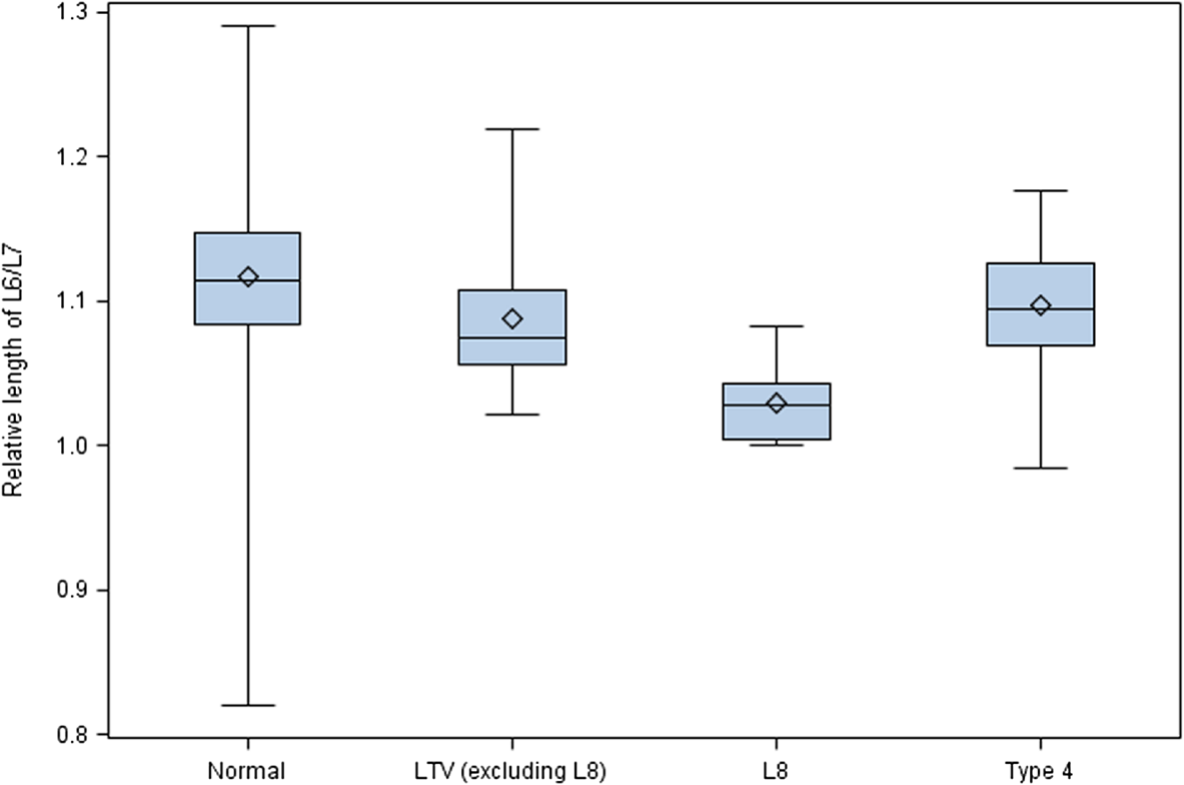
**A box plot of mean relative length of sixth and seventh lumbar vertebrae (L6/L7) in 228 German shepherd dogs with median (horizontal line), mean (square), upper and lower quartiles (box), sample maximum (upper whisker) and sample minimum (lower whisker).** Normal = normal lumbosacral junction, LTV = lumbosacral transitional vertebra of types 1–3, L8 = eighth lumbar vertebra, Type 4 = separation of S1 spinous process from the median crest of the sacrum.

Of the 228 dogs, 217 (95.2%) had seven, ten dogs (4.3%) had eight, and one dog with LTV (0.4%) had six lumbar vertebrae. The caudal position of the L7 in relation to the ilium was the most common type. All dogs with L8, but none of the dogs with normal lumbosacral junctions had cranial position of the L7 in relation to the ilium (Figure 12). Clear differences were detected when comparing the length of L6/L7 between dogs with all types of LTV and dogs with normal lumbosacral junctions (Figure 13). The proportion of dogs belonging to the group with L8 compared to the group with normal lumbosacral junction was 14.2-fold higher (95% CI 3.41 - 59.4) when the relative length of L6/L7 decreased by 0.1 units. The difference between LTV and type 4 against normal junctions was also significant, but a bit smaller, odds ratios were 3.14 (95% CI 1.23 - 8.01) and 2.18 (95% CI 1.17 - 4.05), respectively; i.e., the longer the L7 in relation to the L6, the greater the probability of LTV. Type 4 did not differ from the other types of LTV in the relative length of L6/L7. The mean relative length of L7/L8 was 1.255 (standard deviation 0.043).

**Figure 14:**
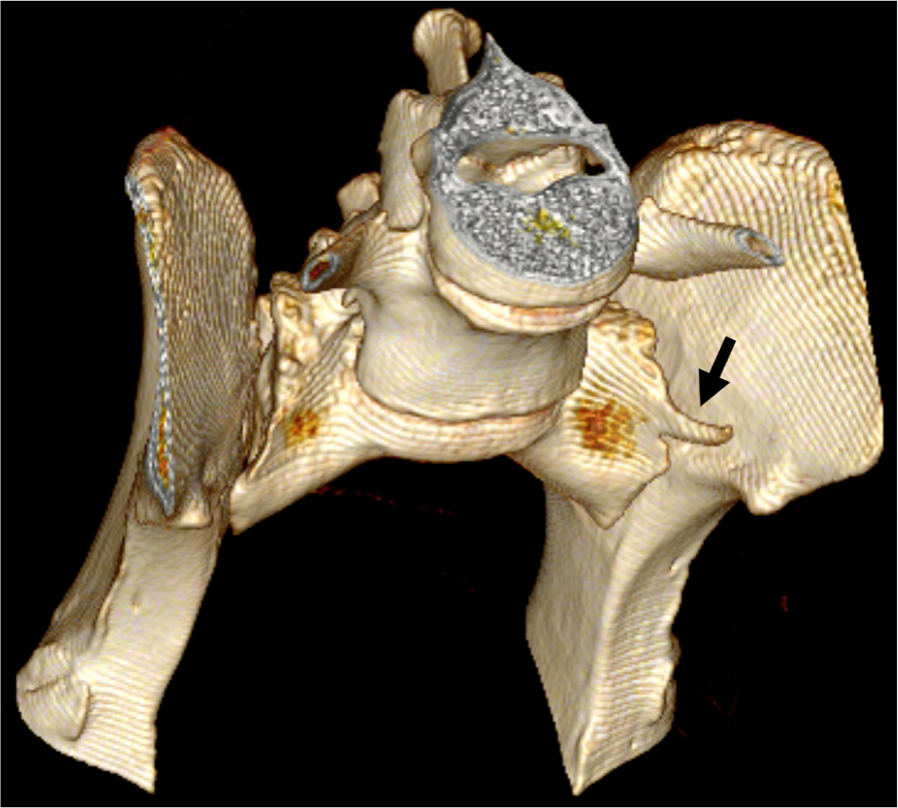
**A CT volume-rendering technique image of a German shepherd dog radiographically diagnosed as type 4.** A small transverse process on the left side, not visible in the radiographs, is seen (arrow).

A CT study was performed in 16 dogs, of which nine dogs had abnormal sacrum and seven were normal. The abnormal findings are presented in Table 2. With CT, the shape of the transverse processes and median crest of the sacrum were easily identified (Figures 9–11). Separation of the S1 spinous process from the median crest of the sacrum seen in the ventrodorsal radiographs (Figure 3) appeared as a deep indentation in the median crest (Figure 9) in CT. One dog classified as type 4, based on radiographs (dog 8 in Table 2) was diagnosed as having a small unilateral transverse process (Figure 14). In two dogs (dogs 3 and 5 in Table 2), the classification based on ventrodorsal radiographs would have changed from lumbar to intermediate type.

**Table 2:** Abnormal computed tomography findings of sacrum and their correlation with radiographs in nine German shepherd dogs

Dog no	Computed tomography findings	Radiological diagnosis	VD	LAT
1 and 2	Deep indentation between S1 and S2 spinous processes	Type 4	Type 4	Normal
3	Deep indentation between S1 and S2 spinous processes	LTV	Type 3/3	Normal
	Bilateral transverse processes			
4	Nonfused S2-S3	LTV	Normal	L8, nonfused S2-S3
5	Nonfused S1-S2, bilateral transverse processes	LTV	Type 3/2	Nonfused S1-S2
6	Deep indentation between S1 and S2 spinous processes	Type 4	Type 4	Normal
7	Nonfused S2-S3	LTV	Normal	L8, nonfused S2-S3
8	Deep indentation between S1 and S2 spinous processes	Type 4	Type 4	Normal
	Unilateral left transverse process			
9	Bilateral transverse processes	LTV	Type 2/2	Normal

VD = ventrodorsal radiograph, LAT = laterolateral radiograph, Type 4 = separation of spinous process of S1 from the median crest,

S1-S2 = sacral vertebrae 1–3, LTV = lumbosacral transitional vertebra, L8 = eighth lumbar vertebrae.

## Discussion

We compared ventrodorsal hip and laterolateral lumbar spine projections in the radiological diagnosis of LTV in German shepherd dogs, and scrutinized the radiographic and CT features of the LTV.

The incidence of LTV is dependent on the definition. In the present study, dogs with eight lumbar vertebrae as the only abnormal finding were classified as to having a LTV, which contrasts with the previous studies. It has argued that L8 is a clinically irrelevant finding [6] and hence the diagnosis can reliably be based on ventrodorsal hip radiographs. However, in our study all ten dogs with eight lumbar vertebrae had a short and caudally positioned last presacral vertebra (L8). The angle and disc space of the lumbosacral junction in these dogs resembled the normal condition, but the position of the L8 relative to the ilium was near the position of the normal S1 vertebra. Additionally, L7 was positioned more cranially in relation to the ilium (Figure 7) and the relative length of L6/L7 was smaller in these dogs. Our findings support those in a study of the vertebral canal between dogs with numerical vertebral variation, in which 11 of 20 dogs with L8 vertebra were German shepherd dogs. In that study, the widest diameter of vertebral canal was at the same level in dogs with seven and eight lumbar vertebra, if L8 was assumed as S1 in the latter group [9].

The incidence of LTV was 40% in our study, which was markedly higher than in other studies [6,10]. In our study, separation of the S1 spinous process from the median crest of the sacrum was classified as LTV, which could explain the high incidence, since the aforementioned radiographic sign was found in 62 dogs (27%) in our study. In a recent study [8], in which separation of the S1 spinous process was classified as LTV, the incidence was also quite high (29%).

In our study, the relative length of L6/L7 was similar in dogs with separation of the S1 spinous process from the median crest as the only abnormality and dogs with LTV, suggesting similar morphology. From the results, we can also conclude that in all three LTV types the relative length of L6/L7 tended to be smaller than in the dogs with normal lumbosacral junctions; i.e. dogs with all types of LTV have a longer L7 in comparison to L6 than dogs with normal lumbosacral junctions. Separation of the S1 spinous process from the median crest as the only abnormality can be genetically a mild form of LTV, and selection against the trait could decrease the incidence of more serious forms of LTV in German shepherd dogs.

The laterolateral projection made the diagnosis of L8 possible. A statistically significant increase in the diagnostic accuracy of LTV was detected when the laterolateral lumbar spine projection was included in the protocol in addition to the ventrodorsal projection. It would have been be possible to diagnose a short L8 with abnormally small transverse processes from the ventrodorsal projection used in hip dysplasia screening, but this would have been difficult if the second to the last lumbar vertebra was not included in the radiograph for comparison. This is seldom the case, because only the last lumbar vertebra is consistently seen in hip radiographs made according to the protocol of Fédération Cynologique Internationale (FCI) [12].

 Some of the radiographic signs, such as separation of the S1 spinous process from the median crest of the sacrum and separation of the sacral vertebrae were visible in only one of the two projections. Every missed case in the ventrodorsal projection had eight lumbar vertebrae. Surprisingly, five of six dogs with symmetrical intermediate or lumbar-type LTV had a normal sacrum based on a laterolateral radiograph. The existence of abnormal transverse processes was ensured with CT in two of these dogs (dog 3 and dog 9 in Table 2). In CT images, a deep indentation between the first and second spinous process of the median crest (Figure 9) was seen in five dogs. In ventrodorsal radiographs this was seen as separation of the S1 spinous process from the median crest of the sacrum (Figure 3). We showed that variation in the radiographic findings of LTV was wide even in the rather small number of cases. The dogs were classified into intermediate (type 2) or sacral (type 3) types, based on visibility of the transverse process in the ventrodorsal radiographic projection. This classification, based on the appearance of the tip of the transverse process, was factitious, since the visibility of the transverse processes of the LTV in radiographs was influenced by the projection and superimposition of the ilium. In Switzerland, the Swiss Dysplasia Committee recently introduced a four-scale grading, in which type 0 is a normal lumbosacral area, type 1 is a sacrum with S1 separated from the median crest of the sacrum, type 2 is a symmetrical LTV, and type 3 is an asymmetrical LTV [13]. This grading was not published when we planned our study, but it seems reasonable, since it does not attempt to classify the LTV, based on the appearance of the transverse processes, which can lead to erroneous classification, as was seen in our study.

A limitation in our study was the lack of radiographs of the total spine, since it can be argued that a transitional vertebra in the thoracolumbar junction can cause the extra vertebra. However, the markedly caudal position of L8 speaks for an LTV. Another limitation was the low number of dogs; however, the results were statistically significant.

## Conclusions

We conclude that laterolateral radiographs of the lumbar spine would be a valuable addition in screening for LTV in German shepherd dogs. The laterolateral projection may, in addition, assist in screening for lumbosacral osteochondrosis and lumbosacral stenosis, two typical conditions in German shepherd dogs. We suggest that L8 be included in the LTV complex in the screening programs. Further studies are needed to elucidate the genetic basis for the different types of lumbosacral and sacral congenital malformations.

## Competing interests

The authors declare that they have no competing interests.

## Authors’ contributions

**AKL** partly collected and analysed all the data and drafted the manuscript

**RS** partly collected and analysed the data

**JJ** performed the statistical analyses and revised the statistical sections of the manuscript

**MS** participated in the design of the study and revision of the manuscript

**OL-V** participated in the design of the study, helped to draft the manuscript, and revised it

All authors read and approved the final manuscript.
